# Informed Consent Prior to Coronary Angiography in a Real World Scenario: What Do Patients Remember?

**DOI:** 10.1371/journal.pone.0015164

**Published:** 2010-12-20

**Authors:** Aslihan Eran, Erland Erdmann, Fikret Er

**Affiliations:** Department of Internal Medicine III, University of Cologne, Cologne, Germany; University of Turin, Italy

## Abstract

**Background:**

Patients' informed consent is legally essential before elective invasive cardiac angiography (CA) and successive intervention can be done. It is unknown to what extent patients can remember previous detailed information given by a specially trained doctor in an optimal scenario as compared to standard care.

**Methodology/Principal Findings:**

In this prospective cohort study 150 consecutive in-patients and 50 out-patients were included before elective CA was initiated. The informed consent was provided and documented in in-patients by trained and instructed physicians the day before CA. In contrast, out-patients received standard information by different not trained physicians, who did not know about this investigation. All patients had to sign a form stating that enough information had been given and all questions had been answered sufficiently. One hour before CA an assessment of the patients' knowledge about CA was performed using a standard point-by-point questionnaire by another independent physician. The supplied information was composed of 12 potential complications, 3 general, 4 periprocedural and 4 procedural aspects.

95% of the patients felt that they had been well and sufficiently informed. Less than half of the potential complications could be remembered by the patients and more patients could remember less serious than life-threatening complications (27.9±8.8% vs. 47.1±11.0%; p<0.001). Even obvious complications like local bleeding could not be remembered by 35% of in-patients and 36% of out-patients (*p* = 0.87). Surprisingly, there were only a few knowledge differences between in- and out-patients.

**Conclusions:**

The knowledge about CA of patients is vague when they give their informed consent. Even structured information given by a specially trained physician did not increase this knowledge.

## Introduction

Today's medical postulate is that a person gives informed consent for a diagnostic or therapeutic intervention only if she or he is competent to act, receives a thorough disclosure about the procedure, comprehends information given and acts voluntarily [1]. While most elements of this universal definition can be controlled by the physician, the quantity and quality of information the patient receives is difficult to assess. Several studies suggest that the components of information are often not successfully communicated owing to poor disclosure on the part of the physician or a lack of patient understanding [2]–[5].

To reform the quality of communication strategies with modified consent forms containing graphics, improved readability, processability, extended discussions, teaching aids, and using video and computer technology have been explored [6]–[10]. In fact, the quality of informed consent is regularly discussed at law courts when undesirable side-effects of medical interventions occur. While physicians try to substantiate the obtained written informed consent by documentation, patients often argue that they have not at all or not sufficiently been informed. Informed consent is more than a legal document, its main aims are to respect and promote patients' autonomy and protect them from potential harm. It is important that the patient understand the diagnosis, indication and purpose of the intervention, prognosis, nature, alternatives, risks, and benefits [11].

Invasive cardiac catheterization and invasive coronary angiography (CA) are the gold standards to diagnose relevant coronary artery disease [12], [13]. For elective catheterization, patients with stable angina pectoris give their written informed consent usually the day before the procedure. In Germany a commercial standard patient information form with graphical illustrations in the course of a 10 – 20 minute conversation is usually used by most physicians to inform the patient.

The goal of the present study was to find out possible knowledge differences between optimally informed in-patients and regularly informed out-patients immediately before CA was to be performed.

## Methods

### Ethics Statement

The study complies with the Declaration of Helsinki, the local ethics committee has approved the research protocol.

### Subjects

**Figure 1:**
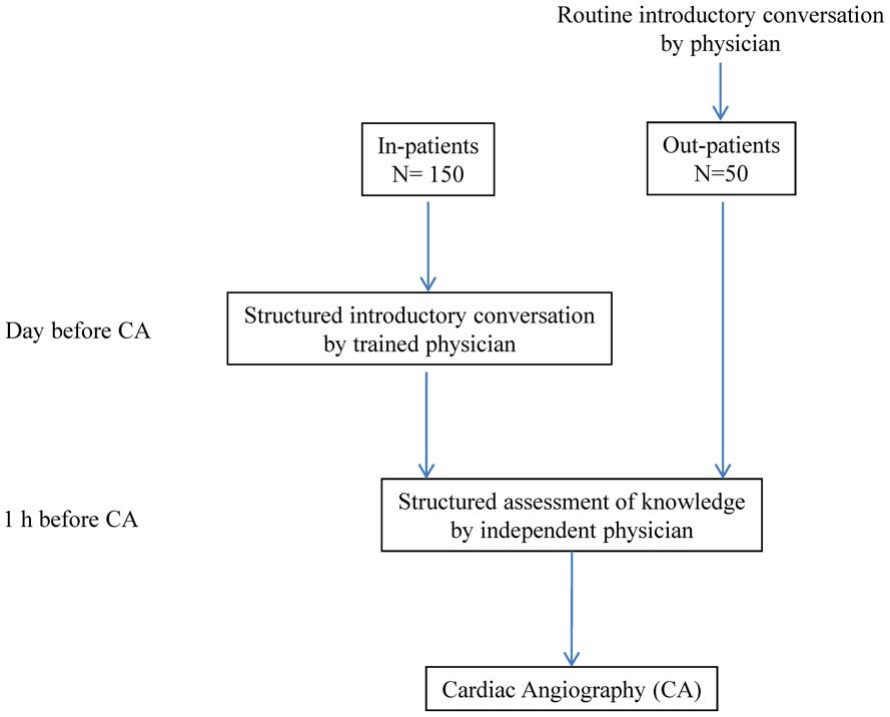
Study flow-chart.

Consecutive patients with stable angina pectoris who were referred to the Cardiology Department of the University Hospital of Cologne for cardiac catheterization were included. Inpatients were admitted the day before the procedure and discharged the day thereafter. Outpatients were admitted on the procedural day and discharged the same or next day. 200 consecutive patients were included; there were no specific exclusion criteria (Figure 1). Patients' education was graded in low (no graduation or less than 9 school-years), middle (high school) and high (college, university).

### Obtaining the informed consent and assessment of knowledge

An advisory board determined the information patients should receive before giving their informed consent. The advisory board recommended that the introductory conversation had to take at least 10 minutes and contain at least the predefined information, which were composed of general information and three topics: pre- and post-procedural rules (fasting 12 hours before CA, immobilization in bed for 12 hours after CA, compression of the groin with a sand bag for 4 hours after CA), information about the catheterization itself (performing CA through femoral artery, use of contrast medium, performing percutaneous intervention (PCI) with either balloon dilatation and/or stenting) and a list of 12 potential complications (trauma of the arteries-venes-nerves, infection, bleeding, allergic reaction, shock, thrombosis, embolism, stroke, myocardial infarction, rescue surgical interventions, cardiopulmonary resuscitation, death (Table 1)). Accordingly, the physicians were trained to optimize their conversation before the first in-hospital patient was included. Doctors were instructed to mention all of the predefined important information.

**Table 1:** Facts listed in the standard questionnaire for assessment of the patients' knowledge.

	General Information
G1	Did the patient get **any information** about the procedure?
G2	Did the patient feel to be **well informed**?
G3	Did the patient have the option to **ask questions**?

	Periprocedural rules
Peri1	Is the patient informed for **fast for** 12 hours before the procedure?
Peri2	Is the patient informed about postprocedural **immobilization** for 12 hours?
Peri3	Is the patient informed about the necessity of **groin compression** with a sand bag after the procedure?
Peri4	Is the patients informed why **aspirin** and **clopidogrel** are administered before catheterization?

	Procedure
Pro1	Is the patient informed about **femoral artery access**?
Pro2	Is the patient informed about the use of **contrast medium**?
Pro3	Is the patient informed about the necessity of **fluoroscopy**?
Pro4Pro4aPro4bPro4c	Is the patient informed about facultative **percutaneous interventions**?AngioplastyStentingAngioplasty+Stenting

	Potential Complications
C1	Trauma of the arteries, venes and nerves
C2	Bleeding
C3	Infection
C4	Allergic reaction
C5	Thrombosis
C6	Embolism
C7*	Myocardial infarction
C8*	Shock
C9*	Cerebral insult
C10*	Emergency operation
C11*	Cardiopulmonary resuscitation
C12*	Death

Asterisk marks serious complications.

The introductory conversation of out-patients was obtained by non-trained general practitioners or external cardiologists. The informing doctors were completely blinded and did not know anything about the present investigation.

From in-hospital patients the informed consent was obtained until 9 p.m. the day before CA. Outpatients gave their informed consent at least 5 days before the procedure. Both, patients and informing physicians signed the documentation chart.

The knowledge of the patients was measured about 1 hour before CA by an independent physician who had not taken part in the information procedure. A standard point-by-point questionnaire was used without the insight of the patient (Table 1).

### Statistical Analysis

Continuous variables are expressed as means ± standard deviation. Comparison of 2 means was performed with the *t* test for normally distributed variables and the Mann-Whitney *U* test for non-Gaussian variables. All statistical tests were 2-tailed, and *p*<0.05 was considered statistically significant.

## Results

### Baseline and general characteristics

A total of two-hundred patients underwent elective coronary angiography (CA). The mean age of all participants was 64.4±12.2 years, out-patients were slightly younger than in-patients (63.2±9.7 years versus 64.8±13.0 years; *p* = 0.43). 118 patients (59%) were men (Table 2). In more in-patients CA had been previously performed than in out-patients (57% vs. 38%; *p* = 0.03). The education and annual income were similar in in- and out-patients (Table 2). More in-patients than out-patients had a known coronary artery disease (56.7% vs. 38.0%; *p* = 0.03). The complete baseline characteristics are listet in table 2.

**Table 2:** Baseline characteristics of in- and out-patients.

	AllN = 200	In-patientsN = 150	Out-patientsN = 50	*p*
**Men/women (%)**	119/81 (60/40)	93/57 (62/38)	26/24 (52/48)	0.25
**Age**	64.4±12.2	64.8±13.0	63.2±9.7	0.43
**Education (%)**LowMiddleHigh	51 (25.5)115 (57.5)34 (17.0)	41 (27.3)86 (57.3)23 (15.3)	10 (20.0)29 (58)11 (22)	0.200.500.12
**Annual income**<30 000 €30 000 – 60 000 €>60 000 €	31 (15.5)119 (59.5)50 (25.0)	24 (16.0)92 (61.3)34 (22.7)	7 (14.0)27 (54.0)16 (32.0)	0.470.230.13
**Medical history**HypertensionDiabetes mellitusCoronary artery diseaseCongestive heart failurePeripheral artery disease	127 (63.5)31 (15.5)104 (52)25 (12.5)10 (5.0)	90 (60.0)21 (14.0)85 (56.7)20 (13.3)8 (5.3)	37 (74.0)10 (20.0)19 (38.0)5 (10.0)2 (4.0)	0.090.370.030.631.00
**Previous catheterization (%)**	104 (52)	85 (57)	19 (38)	0.03
**Language capability (%)** **First language** **Good** **Moderate** **With translator**	171 (86)10 (5)15 (8)4 (2)	132 (88)8 (5)9 (6)1 (1)	39 (78)2 (4)6 (12)3 (6)	0.05

During reassessment 2 patients (1%; 1 in-patient, 1 out-patient; *p* = 0.44) argued that they had not been informed at all about the procedure (Table 2). 95% of the patients (95% in-patiens, 94% out-patients, *p* = 1.00) found the quality of the given information to be sufficient, 5% had expected more details. Most of the patients (95%, 94% in-patients, 96% out-patients; *p* = 0.73) felt that they had enough time and ample opportunity to ask questions.

### Knowledge about periprocedural behavior

Four facts assessed the knowledge of periprocedural rules: patients were not allowed to eat or drink for 12 hours before the procedure (fasting), they should lie supine for 12 hours after the procedure (immobilization), they should use a sand bag for compression of the groin at least for 4 hours after CA and take aspirin and a loading dose of clopidogrel before CA (Table 3).

**Table 3:** Positive answers of the patients just before the coronary angiography.

	AllN = 200	In-patientsN = 150	Out-patientsN = 50	*P*
**General information**				
G1 - Any information (%)	198 (99)	149 (99)	49 (98)	0.44
G2 - Well informed (%)	189 (95)	142 (95)	47 (94)	1.00
G3 - Questions (%)	189 (95)	141 (94)	48 (96)	0.73
**Periprocedural information**				
Peri1 - Fasting (%)	178 (89)	129 (86)	49 (98)	0.018
Peri2 – Immobilization (%)	172 (86)	135 (90)	38 (76)	0.017
Peri3 - Compression (%)	168 (84)	128 (85)	40 (80)	0.38
Peri4 - ASS/Clopidogrel (%)	104 (52)	89 (59)	15 (30)	0.001
**Procedural information**				
Pro1 - femoral access (%)	193 (97)	147 (98)	46 (92)	0.07
Pro2 - contrast medium (%)	188 (94)	142 (95)	46 (92)	0.50
Pro3 - fluoroscopy	120 (60)	84 (56)	36 (72)	0.048
Pro4 – PCIPro4a – Angioplasty (%)Pro4b – Stenting (%)Pro4c – Angioplasty+Stenting (%)	17 (9)14 (7)169 (85)	15 (10)11 (7)124 (83)	2 (4)3 (6)45 (90)	0.38

Provided and assessed information were composed of general (G1-G3), periprocedural (Peri1-Peri4) and procedural (Pro1-Pro4) facts (see table 1).

129 in-patients (86%) and 49 out-patient (98%) did know that they should fast before CA (*p* = 0.018). More in-patients (n = 135; 90%) than out-patients (n = 38; 76%) remembered that they had to be immobilized after CA (*p* = 0.017). The need for arterial compression with a sand bag after CA was known by 168 (84%) of all patients (128 (85%) of in-patients vs. 40 (80%) of out-patients; *p* = 0.38). Only 104 patients (52%) knew the indication for aspirin and clopidogrel: 89 in-patients (59%) vs. 15 out-patients (30%; *p* = 0.001).

### Knowledge about CA procedure

The knowledge about CA was assessed by four facts: access via femoral artery, the use of contrast medium and X-radiation during examination and the feasibility for PCI.

That CA would be performed via femoral artery was known by 193 patients (97%), 147 in-patients (98%) and 46 out-patients (92%; *p* = 0.07). The necessity of contrast medium use was known by 188 patients (94%), 142 in-patients (95%) and 46 out-patients (92%; *p* = 0.50).

Only 120 patients (60%) did know that X-radiation will be used for fluoroscopy: 84 in-patients (56%) vs. 36 out-patients (72%; *p* = 0.048). The possibility of PCI with angioplasty and stenting was known by 169 patients (85%), 124 in-patients (83%) vs. 45 out-patients (90%; *p* = 0.38).

### Knowledge about potential complications

**Figure 2:**
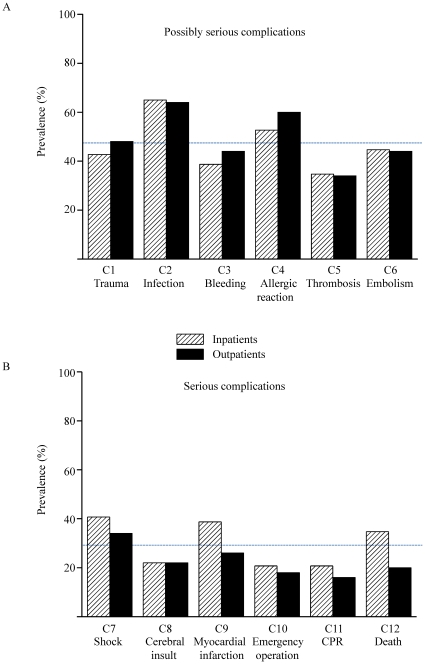
Knowledge of the patients in respect to potential complications. A, possible serious complications; B, serious complications. Dashed line mark the average frequency of remembered complications.

Potential complications were divided in two categories: possibly serious complications (trauma of vessels or nerves, bleeding, infection, allergic reaction, thrombosis and embolism) and life-threatening complications (myocardial infarction, shock, cerebral insult, emergency operation, cardiopulmonary resuscitation and death). The frequency of recalling possibly serious complications and/or life threatening complications were statistically not different in in-patients vs. outpatients (Figure 2). Overall more patients could remember less serious than life-threatening complications (47.1±11.0% vs.27.9±8.8%; *p*<0.001).

## Discussion

In the present study we simulated an optimal life scenario, in which patients undergoing CA gave their informed consent after a structured informing conversation with a trained physician. The comparative group was composed of out-patients who had been informed several days earlier and not systematically. Patients were consecutively included in this study without exclusion of any patient. This investigation should reflect real life irrespective of patients' individual characteristics like education or occupation. We had expected that a structural assessment of the patients' knowledge would reveal that in-patients could reproduce more facts than out-patients. Surprisingly, only a few differences in knowledge were observed concerning the general, peri- and procedural information between the two groups. Actually, significantly more out-patients than in-patients remembered that they had to fast before the procedure and that fluoroscopy would be used during CA.

Complications were remembered rather infrequently. Interestingly, less serious complications were better remembered than life-threatening serious ones. Even obvious complications like bleeding or puncture related trauma was known by less than 50% of the patients. Considering that half of the patients had undergone previous CA, the reported lack of knowledge is alarming. 2 patients (1%) even affirmed that they had not been informed at all.

Patients' informed consent is essential ethically and legally before physicians are allowed to act [14]. It is a challenge, especially in court for doctors to prove retrospectively what they told the patients. In some cases the provided information might be insufficient and incomplete, but we see even in what we consider an optimal setting that a high percentage of the patients do not remember fundamental and essential facts they were certainly told one day before CA, although 95% of them had felt to be sufficiently informed and had the opportunity to ask further questions. Out-patients were informed by several and different not specially trained physicians. However their knowledge was not inferior compared to in-patients.

We are not able to explain these observations. A recent meta-analysis of surgical patients confirms that patients are often not able to understand the provided information and recall these later [15]. We can only speculate, whether the provided facts are too technical or too special and reflect the modern high-tech medicine or whether patients trust physicians and their good work so much that they do not see the necessity to remember any possible adverse events.

Another question to address is the potentially reduced mental capacity of patients undergoing a complicated procedure. That Alzheimer's disease, other dementia and some psychiatric disorders may limit the mental capacity of patients is understandable [16]–[20]. There were no patients with these diagnoses in our cohort. The relevance of several medical disorders like infections, cancer or coronary artery disease is not clear and controversially discussed [21]–[24]. The observation that less serious complications were better remembered than serious ones highlight that is not only a matter of mental capacity why patients forget what we consider very relevant information.

There are currently no formal practice guidelines from professional societies for the assessment of the capacity of patients to consent to treatment [14]. We see a large area of uncertainty how to handle this daily problem. A major evolution in the field of medicine over the past decades was the transition from a paternalistic way of approaching patients to a shared decision-making process. We should constantly be aware of the fact that many of our patients inspite of what we believe was good and thorough information, cannot recall this later on. Their interest is to get help and not to be troubled with the many possible problems involved. If by chance a serious complication occurs, they probably “honestly” state that they have never been informed about this before the procedure.
